# Magnetic resonance imaging characteristics in patients with histopathologically proven fibrous dysplasia—a systematic review

**DOI:** 10.1007/s00256-020-03388-x

**Published:** 2020-02-10

**Authors:** Anna-Reetta Kinnunen, Reijo Sironen, Petri Sipola

**Affiliations:** 1 Institute of Clinical Medicine, University of Eastern Finland, Yliopistonranta 1, 70210 Kuopio, Finland; 2 Department of Clinical Pathology, Kuopio University Hospital, Kuopio, Finland; 3 Department of Clinical Radiology, Kuopio University Hospital, Kuopio, Finland

**Keywords:** Fibrous dysplasia, MRI, Histopathology, Diagnostics, Review article

## Abstract

**Objective:**

To examine the demographics, lesion location, and characteristic magnetic resonance imaging (MRI) findings in patients with histopathologically proven fibrous dysplasia (FD).

**Materials and methods:**

A systematic literature search of the MRI findings in patients with histologically proven FD was performed. Altogether, 76 articles with 136 patients were evaluated.

**Results:**

The mean age of the patients was 35.0 + − 18.5 years (range 1 month–75 years). Fifty-eight of the cases were females, 51 males, and in 27 gender was not defined. The most common locations were craniofacial (*n* = 55 (40%)), long bones (*n* = 31 (23%)), and spine (*n* = 24 (18%)). The monostotic form of FD was the most common. Signal intensities (SI) on T1-weighted images were predominantly hypointense (*n* = 46 (37%)). The SI was highly variable on T2-weighted images with hyperintensity being most common (*n* = 22 (18%)). Contrast enhancement was found in 75 (55%) FD patients. Secondary aneurysmal bone cysts (ABCs) and malignant transformation in patients without prior radiotherapy was found in some patients.

**Conclusion:**

Current knowledge of the MRI findings in patients with FD is based mainly on case reports. SI in patients with FD is variable and contrast enhancement is common. FD may explain etiology of spinal bone tumor in some patients. FD with malignant transformation should be considered also in patients without prior radiotherapy. Further studies are needed to clarify if FD displays specific characteristics allowing it to be distinguished from other bone tumors.

## Introduction

Fibrous dysplasia (FD) is a benign developmental disorder of bone; different sizes of lesions can occur on either one (monostotic form) or multiple bones (polyostotic form). FD manifests in tumor-like lesions in bone, where connective tissue and immature bone replaces the normal bone structure [1]. The most common sites for the lesions are long bones, ribs, and craniofacial bones though lesions may occur throughout the skeleton; instead, the involvement of spine, hand, and feet is rare [1]. The pathophysiology of the diseases is connected to a mutation in the guanine nucleotide binding, an alpha stimulating (GNAS1) gene that encodes the stimulatory guanine nucleotide–binding protein Gs alpha which causes defects in osteoblastic differentiation. It is estimated that FD accounts for 5 to 7% of all benign bone tumors and it is one of the diagnosis commonly discussed when differentiating between different tumorous bone lesions [1, 2].

Histopathologically, FD is usually a well-defined, tan-gray mass [3]. In FD, the presence of bone trabeculae is the reason for the development of structures with dense and variably fibrous so-called gritty qualities. Particularly in older FD lesions, there might be prominent cyst formation [3]. Occasionally, chondroid metaplasia provides the lesion with a glassier, blue-tinged appearance. Varying proportions of fibrous and osseous tissue are present in the lesions. Irregular, curvilinear, trabeculae of woven bone (rarely lamellar bone) is arranged in a pattern that resembles letters in the Chinese alphabet. The osseous component typically lacks osteoblastic rimming. Fibrous stroma is low to moderately cellular. The chondroid component, aneurysmal bone cyst (ABC)–like changes, foam cells, giant cells, and a myxoid change may appear as a secondary disturbance in FD. The overall appearance of the lesion is bland, and it lacks cytologic atypia [3].

The appearance of FD in magnetic resonance imaging (MRI) scans has not been widely investigated. Though some researchers claim that FD has a characteristic appearance in MRI, there are also reports indicating that the diagnosis may only be confirmed after a biopsy [4–7]. The estimated prevalence of malignant transformation in FD ranges from 0.4 to 6.7% [8]. In radiographs, FD-related malignancies have been described as mineralized, poorly marginalized osteolytic lesions with cortical destruction, though these characteristics may also be present in a benign FD lesion [8]. Due to the increasing popularity of MR imaging, the MRI characteristics of FD should be more familiar to radiologists [9]. The purpose of this review is to examine the demographics, tumor location, and characteristic MRI findings in patients with histopathologically proven FD.

## Materials and methods

**Fig. 1:**
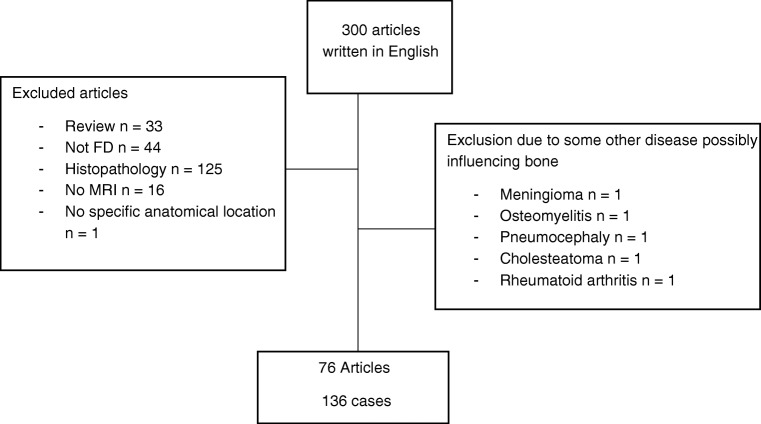
Flowchart of the study

In August 2019, a systematic literature search was conducted in the PubMed database combining MeSH terms “fibrous dysplasia of the bone” and “magnetic resonance imaging.” We accepted those studies with histologically proven FD that had histopathological diagnostic criteria described in the article, MRI from the area of the FD lesion, and no overlapping pathology in the lesion area. Initially, the search identified 369 articles, of which 300 were written in English. Of those 300, 224 (75%) were excluded. The reasons for exclusions were as follows: 33 (11%) articles were review studies with no patient cases and 44 (15%) articles considered FD only as a differential diagnosis for some other disease. There were 125 (42%) articles that lacked the histopathological confirmation or criteria (e.g., only mentioned that the case was biopsy proven but had no mention about the histopathology of the disease) or had multiple patient cases with part being histopathologically diagnosed but not differentiated from those for whom there was no histopathological confirmation. Sixteen articles (5%) lacked details of the MRI results from the FD area or the MRI characteristics were not described in the article. In one article (0.3%), the anatomical location of the lesions was not specified, and therefore, this publication was excluded [10]. Five articles (2%) presented FD with a coexisting pathology and were excluded as the pathology was interrelated with the FD lesion. Coexisting pathologies in patients with craniofacial FD were meningioma, osteomyelitis, pneumocephaly, and cholesteatoma. One article described a patient case with femoral FD and rheumatoid arthritis. The final number of articles included was 76 (25%). Figure 1 shows the flowchart of the included and excluded articles and reasons for exclusion.

Some articles contained multiple cases but only some of them were histopathologically proven and had MRI taken from the area of the lesion. From those articles, we only accepted the cases that fulfilled the inclusion criteria.

The number of included patients in the articles varied from 1 to 16. The vast majority, i.e., 66 out of 76 articles (87%), were case reports investigating only one study subject. There were 6 (9%) articles with 2 to 5 study subjects and 6 to 10 patients in two articles (3%), and two articles (3%) had examined more than 10 patient cases. In patients with polyostotic FD, one patient could be present in more than one anatomical category. Lesions in sacrum were categorized as vertebral FD. One monostotic lesion was covering both the rib and vertebra areas but was categorized as costal FD because of the more extensive involvement of the rib.

MRI signal intensities (SI) and contrast enhancement patterns with gadolinium-based contrast medium were tabulated. A sub-division into epiphyseal, metaphyseal, or diaphyseal location was made in 8/31 (26%) and a sub-division into centric or eccentric in 4/31 (13%) of FD lesions in long bones. The location of the phalangeal FD lesion was described as diaphyseal [11]. The sizes of the FD lesions were described in 13/136 (10%) cases.

## Results

### Demographics and location of the lesion

The mean age of the patients was 35.0 + − 18.5 years (range 1 month–75 years); 58 of them (43%) were females and 51 males (38%); in 27 (20%) cases, the patient’s gender had not been mentioned. The location of FD in descending order was as follows: craniofacial (*n* = 55 (40%)), long bones (*n* = 31 (23%)), spine (*n* = 24 (18%)), pelvis (*n* = 7 (5%)), ribs (*n* = 5 (4%)), phalanges (*n* = 1, 0.7%), and metacarpal (*n* = 1 (0.7%)). Malignant transformation was presented in 12 (9%) study subjects. Eighty of the cases (59%) were monostotic, and 39 (29%) were polyostotic, and in 17 (13%) cases, no differentiation between monostotic and polyostotic forms had been made. Polyostotic FD was most common in long bones (*n* = 16) where it was more common than monostotic FD (*n* = 14). A summary of the study subjects and the location of FD is presented in Table 1.

**Table 1:** Summary of cases included in the review

Category	Craniofacial	Long bones	Vertebra	Pelvis	Rib	Phalanx	Metacarpal	Malignant transformation	Total
Mean age	28.3 + − 18.4	30.7 + − 19.4	40.6 + − 26.4	42.0 + − 26.4	50.2 + − 12.7	14	15	48.0 + − 7.7	35.0 + − 18.5
Age not mentioned	17 (31%)	8 (26%)	2 (8%)	- (0%)	- (0%)	- (0%)	- (0%)	4 (33%)	31
Female	22 (40%)	13 (42%)	10 (42%)	6 (86%)	2 (40%)	- (0%)	- (0%)	5 (42%)	58
Male	16 (29%)	10 (32%)	12 (50%)	1 (14%)	3 (60%)	1 (100%)	1 (100%)	7 (58%)	51
Sex not mentioned	17 (31%)	8 (26%)	2 (8%)	- (0%)	- (0%)	- (0%)	- (0%)	- (0%)	27
Monostotic	32 (58%)	14 (45%)	14 (58%)	6 (86%)	4 (80%)	1 (100%	1 (100%)	8 (67%)	80
Polyostotic	7 (13%)	16 (52%)	10 (42%)	1 (14%)	1 (20%)	- (0%)	- (0%)	4 (33%)	39
Mono/poly not defined	16 (29%)	1 (3%)	- (0%)	- (0%)	- (0%)	- (0%)	- (0%)	- (0%)	17
Total	55 (40%)	31 (23%)	24 (18%)	7 (5%)	5 (4%)	1 (0.7%)	1 (0.7%)	12 (9%)	136

### MRI signal intensity in all patients with FD without malignant transformation

The summary of MRI SI in all FD patients without malignant transformations is shown in Table 2. On T1-weighted images, the SI in 75/124 (60%) patients was hypointense and/or intermediate. However, in 24/124 (19%) patients, some hyperintense SI was observed on T1-weighted images. On T2-weighted images, the SI was extremely variable such that the proportion of SI was distributed almost evenly in all categories.

**Table 2:** Signal intensities of FD patients without malignant transformation, *n* = 124

	Hypointense	Intermediate	Hyperintense	Hypo+ intermediate	Hypo + hyper	Intermediate+ hyper	Heterogeneous	Not mentioned
T1W	46 (37%)	22 (18%)	3 (2%)	7 (6%)	7 (6%)	5 (4%)	9 (7%)	25 (20%)
T2W	19 (15%)	10 (8%)	22 (18%)	4 (3%)	10 (8%)	15 (12%)	15 (12%)	29 (23%)

*T1W* T1-weighted, *T2W* T2-weighted

Contrast enhancement in all FD patients without malignant transformations is summarized in Table 3. Slightly over half of the patients, 63/124 (51%), showed at least some enhancement.

**Table 3:** Contrast enhancement of FD patients without malignant transformation, *n* = 124

None	Moderate	Heterogeneous	Diffuse	Strong	Soft tissue enhancement	Not mentioned
12 (10%)	17 (14%)	18 (15%)	15 (12%)	10 (8%)	3 (2%)	49 (40%)

### Craniofacial FD

Craniofacial FD presented in 55/136 (40%) of the all FD patients; i.e., it was the most common location of FD; 32 of these patients had monostotic FD and seven had the polyostotic form. In 16 cases, no sub-division into mono- or polyostotic FD had been conducted. Of the patients with craniofacial FD with known gender (*n* = 38), craniofacial FD seemed to be somewhat more common in females (*n* = 22 (58%)) than in males (*n* = 16 (42%)).

Monostotic lesions were located in clivus (9 cases), temporal bone (3 cases), sphenoid bone (3 cases), maxilla (3 cases), mastoid bone (1 case), frontal bone (1 case), and occipital bone (1 case). Other monostotic lesions were located in the ethmoid/frontal sinus (2 cases), in the spheno-ethmoidal area (2 cases), one in the clivus and sphenoid sinus area, one in the sphenoid bone-orbital apex-maxillary sinus area, one in the superomedial compartment of orbita, one in the clivus and occipital bone, one in the orbital roof-frontal bone, one in the orbital roof, frontal, sphenoid, and ethmoid bone area, and one extending from the nasopharyngeal area to the level of the sylvian fissure.

The locations of the polyostotic FD were as follows: one in the clivus, one in the sphenoid bone, one in the clivus, dorsum sellae, and in the condyles and foraminal part of the occipital bone, one in calvarium (sphenoid, parietal and occipital bone), one in the temporal bone, mandibula, and base of the skull, one in the temporal, occipital, parietal, frontal, and sphenoid bone, and one involving all cranial and facial bones.

Sixteen lesions had been identified in the facial area with no further specification and without a sub-division into either monostotic or polyostotic FD.

The SI on T1- and T2-weighted images and contrast enhancement in patients with craniofacial FD is summarized in Tables 4 and 5. A soft tissue enhancement was present in one patient (2%).

**Table 4:** Signal intensities in T1-weighted and T2-weighted MRI of craniofacial FD, *n* = 55

	Hypointense	Intermediate	Hyperintense	Hypo + intermediate	Hypo + hyper	Intermediate + hyper	Heterogeneous	Not mentioned
T1W	16 (29%)	6 (11%)	1 (2%)	4 (7%)	5 (9%)	1 (2%)	3 (5%)	19 (35%)
T2W	15 (27%)	4 (7%)	6 (11%)	3 (5%)	4 (7%)	3 (5%)	2 (4%)	18 (33%)

*T1W* T1-weighted, *T2W* T2-weighted

**Table 5:** Contrast-enhanced MRI of craniofacial FD, *n* = 55

None	Moderate	Heterogeneous	Diffuse	Strong	Soft tissue enhancement	Not mentioned
9 (16%)	12 (22%)	5 (9%)	6 (11%)	5 (9%)	1 (2%)	17 (31%)

### Long bone FD

FD was found in the long bones of 31out of 136 patients (23%), i.e., the long bones were the second most common location of FD. These 31 were almost equally divided into polyostotic (16 cases) and monostotic (14 cases); in one case, no sub-division into mono- or polyostotic forms had been made. Gender was mentioned in 23 (74%) cases, of them 13 (57%) were females and 10 (43%) males.

Of the 31 patients with long bone FD, FD was most commonly found in the lower leg bones: the femur (in 16 (52%)) and tibia (in five patients (16%)). In 10 patients (32%), FD was found in the upper limb (six in humerus, three in radius, and one in ulna). The locations of the lesions had been described as centric in three cases of monostotic FD and eccentric in one polyostotic case. In the other patients, the location of centricity was not mentioned. Lesions were epiphyseal in two cases (both monostotic FD), diaphyseal in four cases (all monostotic FD), and metaphyseal in one case; there was one monostotic lesion located in both diaphyseal and metaphyseal regions of the bone.

Table 6 summarizes the SIs in patients with long bone FD. Cystic areas in long bone FD were relatively common and found in 11/31 (35%) of long bone FD patients. Contrast enhancement was noted in 17/31 (55%) cases. The patterns of enhancement are summarized in Table 7.

**Table 6:** Signal intensities of FD of long bones, *n* = 31

	Hypointense	Intermediate	Hyperintense	Hypo + intermediate	Hypo + hyper	Intermediate + hyper	Heterogeneous	Not mentioned
T1W	14 (45%)	11 (35%)	1 (3%)	1 (3%)	- (0%)	3 (10%)	1 (3%)	- (0%)
T2W	- (0%)	3 (10%)	10 (32%)	- (0%)	1 (3%)	4 (13%)	10 (32%)	3 (10%)

*T1W* T1-weighted, *T2W* T2-weighted

**Table 7:** Contrast enhancement of FD in the long bones, *n* = 31

None	Moderate	Heterogeneous	Diffuse	Strong	Soft tissue enhancement	Not mentioned
- (0%)	2 (6%)	10 (32%)	3 (10%)	1 (3%)	1 (3%)	14 (45%)

Interestingly, in 2/31 patients, soft tissue extension and cortical destruction were noted and one of these cases presented with soft tissue contrast enhancement.

### Vertebral FD

Vertebral FD was not uncommon, being evident in 24/136 (18%) of all the patients with FD. In 14/24 (58%) patients, vertebral FD was monostotic and in 10/24 (42%) polyostotic. Of these 24 patients, 10 (42%) were female and 12 (50%) were male but the gender was not mentioned in two study subjects (8%).

The location of vertebral FD was cervical in four (17%) patients, thoracic in seven (29%) patients, lumbar in seven (29%) patients, and sacral in two (8%) patients. In two cases (8%), FD involved cervical, thoracic, and lumbar spine and in two cases (8%), the location was thoracic or lumbar spine. The location within the vertebra was the vertebral body in eight (33%) patients, posterior elements in one (4%) patient, sacral ala in one (4%) patient, sacral body and ala in one (4%) patient, vertebral body and pedicle in one (4%) patient, vertebral body and posterior elements in five (21%) patients, and vertebral body, pedicle, and posterior elements in six (25%) patients. In one (4%) patient, the location within the vertebra was not mentioned.

SI on T1- and T2-weighted images of vertebral FD are presented in Table 8. The types of contrast enhancement are summarized in Table 9. A pathological fracture was not a rare event, being found in six patients (25%) with vertebral FD.

**Table 8:** Signal intensities of vertebral FD, *n* = 24

	Hypointense	Intermediate	Hyperintense	Hypo + intermediate	Hypo + hyper	Intermediate + hyper	Heterogeneous	Not mentioned
T1W	14 (58%)	1 (4%)	- (0%)	2 (8%)	2 (8%)	- (0%)	1 (4%)	4 (17%)
T2W	4 (17%)	1 (4%)	4 (17%)	1 (4%)	5 (21%)	6 (25%)	- (0%)	3 (13%)

*T1W* T1-weighted, *T2W* T2-weighted

**Table 9:** Contrast enhancement of vertebral FD, *n* = 24

None	Moderate	Heterogeneous	Diffuse	Strong	Soft tissue enhancement	Not mentioned
3 (13%)	3 (13%)	3 (13%)	4 (17%)	4 (17%)	- (0%)	7 (29%)

### Pelvic FD

FD in pelvis is relatively rare, being present in 7/136 (5%) of all the FD patients. Six out of the seven patients with pelvic FD were females. The form of the FD was monostotic in six and polyostotic in one patient.

The SIs in the MRI scans are shown in Table 10. Contrast enhancement was poorly studied and had been reported in one patient only. Contrast enhancement was described as a diffuse soft tissue enhancement [12]. Cortical destruction was also present in this aforementioned case.

**Table 10:** Signal intensities of pelvic FD, *n* = 7

	Hypointense	Intermediate	Hyperintense	Hypo + intermediate	Hypo + hyper	Intermediate + hyper	Heterogeneous	Not mentioned
T1W	1 (14%)	4 (57%)	- (0%)	- (0%)	- (0%)	- (0%)	2 (29%)	- (0%)
T2W	- (0%)	2 (29%)	1 (14%)	- (0%)	- (0%)	1 (14%)	1 (14%)	2 (29%)

*T1W* T1-weighted, *T2W* T2-weighted

### Costal, phalangeal, and metacarpal FD

Costal FD was found in five out of 136 (4%) FD patients. Two of these were females and three were men; in one of the patients, the FD was polyostotic. The SI of costal FD is summarized in Table 11. Contrast enhancement was reported in two patients and both cases reported diffuse enhancement.

**Table 11:** Signal intensities of costal FD, *n* = 5

	Hypointense	Intermediate	Hyperintense	Hypo + intermediate	Hypo + hyper	Intermediate + hyper	Heterogeneous	Not mentioned
T1W	1 (20%)	- (0%)	- (0%)	- (0%)	- (0%)	1 (20%)	2 (40%)	1 (20%)
T2W	- (0%)	- (0%)	1 (20%)	- (0%)	- (0%)	- (0%)	2 (40%)	2 (40%)

*T1W* T1-weighted, *T2W* T2-weighted

Phalangeal FD is rare; it was found only in one out of 136 (0.7%) patients. In this patient, FD was in the diaphysis of the 3rd phalanx. The patient was a 14-year-old male [11]. On T2-weighted image, the SI was described as a mixture of an isointense and a high signal. There was no mention made of T1-weighted images or contrast enhancement.

Metacarpal FD was also found in one patient. The monostotic FD was located in the 2nd metacarpal of a 15-year-old male [13]. In contrast to most lesions, the SI on T1-weighted image was high in this patient and the lesion showed soft tissue extension. T2-weighted images or contrast enhancement was not noted.

### FD with ABC-like changes

Secondary ABC was relatively common and was found in 8/136 (6%) of all patients with FD. Seven of them (88%) appeared to be monostotic; the other one had polyostotic FD. Five of the eight patients (63%) with secondary ABC were males, and three (38%) were females.

Half of the patients (*n* = 4 (50%)) with ABC-like changes had craniofacial FD; two patients (25%) had long bone FD. The other two subjects with ABC-like changes had FD, one in the iliac bone, the other in vertebra and rib.

In MRI scans, the SIs in patients with FD and secondary ABC are shown in Table 12. As suggested, T2 imaging showed at least some hyperintensity in all patients. In all cases, there was some mention of a cystic appearance. Fluid-fluid or fluid level appearance of ABC in MRI was noted in 4/8 (50%) cases. Cortical destruction was found in one patient and there had been a pathological fracture in one patient.

**Table 12:** Signal intensities of FD with ABC-like changes, *n* = 8

	Hypointense	Intermediate	Hyperintense	Hypo + intermediate	Hypo + hyper	Intermediate + hyper	Heterogeneous	Not mentioned
T1W	- (0%)	3 (38%)	- (0%)	- (0%)	2 (25%)	- (0%)	2 (25%)	1 (13%
T2W	- (0%)	- (0%)	2 (25%)	- (0%)	- (0%)	2 (25%)	2 (25%)	2 (25%

*T1W* T1-weighted, *T2W* T2-weighted

Contrast enhancement was reported in two patients. In one patient, the contrast enhancement was reported to be strong; in the other patient, the enhancement was described as a ring enhancement with a peripheral rim around the lesion.

### Malignant transformation

A malignant transformation in the FD lesions was found in 12/136 (9%) patients. Of those patients, eight (67%) had monostotic FD and four (33%) had polyostotic FD. Seven (58%) of the patients were males and five (42%) were females. In eight patients (67%), a malignant transformation was found in the long bones (five in the femur, three in the tibia), three (25%) in the craniofacial area, and one (8%) in the rib.

Different types of malignancies were associated with FD, i.e., osteosarcoma (six cases), fibrosarcoma (two cases), and malignant fibrous histiocytoma (one case). Three cases were classified as sarcoma but without further details. None of the patients had received prior radiotherapy.

The MRI SIs in patients with FD with malignant transformation are shown in Table 13. On T1-weighted images, the SI was predominantly mixed hypointense and intermediate (*n* = 8 (67%)). On T2-weighted images, SI was hyperintense in eight (67%) patients. It was rare that there would be hypointense SI on T2-weighted images in patients with FD with malignant transformation; this was found in only one (8%) patient.

**Table 13:** Signal intensities of FD with malignant transformation, *n* = 12

	Hypointense	Intermediate	Hyperintense	Hypo + intermediate	Hypo + hyper	Intermediate + hyper	Heterogeneous	Not mentioned
T1W	2 (17%)	1 (8%)	- (0%)	8 (67%)	- (0%)	- (0%)	- (0%)	1 (8%)
T2W	1 (8%)	- (0%)	8 (67%)	- (0%)	1 (8%)	- (0%)	- (0%)	2 (17%)

*T1W* T1-weighted, *T2W* T2-weighted

Cortical destruction was common in FD patients with malignant transformations and found in 6 of 12 (50%) patients. It was more common that there would be tumor extension to soft tissue since this was found in 9/12 (75%) patients. The types of contrast enhancement in FD patients with malignant transformation are summarized in Table 14. In all patients, the tumor was enhanced with contrast agent, typically in a heterogeneous manner; this was found in 9 out of 12 (75%) patients.

**Table 14:** Contrast enhancement of FD with malignant transformation, *n* = 12

None	Moderate	Heterogeneous	Diffuse	Strong	Soft tissue enhancement	Not mentioned
- (0%)	1 (8%)	9 (75%)	- (0%)	2 (17%)	- (0%)	- (0%)

Two patients (17%) had biopsy proven benign FD before the malignant diagnosis.

### Other findings

Pathological fractures were evident in 9 (7%) study subjects. The locations of the fractures were vertebra (six benign cases), the femur (two cases with malignant transformation), and humerus (one benign case). Six of the fractures were in patients with monostotic FD and three in patients with polyostotic FD.

Soft tissue extension was present in 15 (11%) patients with FD, nine of these (60%) were FD patients with a malignant transformation. The locations of FD with soft tissue extension and without malignant transformation were as follows: iliac bone (two patients), femur, humerus, vertebra, and metacarpal bone (one each). Two patients without malignant transformation of FD presented with both a soft tissue extension and pathological fractures (one monostotic humeral lesion and one monostotic lesion in Th5 vertebra).

## Discussion

MRI characteristics in patients with histopathologically proven fibrous dysplasia are not comprehensively described in the current literature. To overcome this problem, we summarized data of all published studies describing MRI characteristics in patients with histopathologically confirmed FD. In addition, patients, demographics, and tumor location were reported. The principal findings of the current study were that FD is most commonly found in craniofacial bones, long bones, and spine. The MR signal intensity and contrast enhancement in patients with FD is highly variable. In craniofacial FD, the T2 SI was most commonly relatively low being hypointense or intermediate in most patients. In contrast, SI was at least partly hyperintense on T2-weighted images in 81% of patients with long bone FD. In all, 11/124 (9%) of FD lesions without malignancy showed pathological fractures and/or soft tissue extension. FD with malignant transformation was found in 9% of all these FD patients. We were not able to find any SI characteristics typical for FD with a malignant transformation. It should be noted, however, that malignant transformation was most common in long bones (67%), even though long bones were only the second most common location for FD.

Our study indicates that the diagnosis of FD only with MRI is challenging due to the high variability of the signal in the T2 and contrast-enhanced images.

The T1 signal was predominantly hypointense, intermediate, or combination of these two in 75/124 (60%) of FD patients. However, SI was hyperintense or partly hyperintense in 24/124 (19%) of the patients in the T1-weighted imaging. This finding is at odds with the findings of Norris et al. [14] and Jee et al. [10] who published the results stating that all 26 patients displayed either intermediate or low signal in the T1-weighted imaging. The studies that displayed high SI on T1 images did not report whether this could be due to intralesional fat, hemorrhage, or protein rich cysts. These entities have been shown to demonstrate a high signal in the T1-weighted imaging [15]. A finding of secondary ABC has been previously described [16]. Indeed, it was not uncommon (found in 6%) in our study patients. Spinal FD was relatively common, being present in every fifth patient and it may also cause pathological fractures. FD is one abnormality that should be considered in the work-up of bone lesions that remain stable in the follow-up of spinal MR examinations. FD may be very active in some patients and display characteristics that are considered to be suspicious for malignancy. The possibility of malignancy cannot be excluded in patients with FD. Indeed, two of the patients with FD with a malignant transformation had a previous diagnosis of FD without any suspicion of malignancy. Previous studies have shown that malignant transformation in FD is most common in the craniofacial region [2] but our review suggests that it is most commonly found in long bones.

In line with previous studies, FD lesions may occur throughout the skeleton [1]; the craniofacial, long bones, ribs, and pelvis were the most common locations of this bone disorder [2]. In addition, a finding of FD with soft tissue extension has been previously reported; Jee et al. [10] reported the presence of soft tissue extension in 4/13 of the patients. In agreement with a previous study, monostotic FD was the most common form of FD accounting for 67% of our patients [1]. Interestingly, polyostotic FD was the most common form of FD in long bones.

The major differences in the conclusions of our review compared with previous studies are as follows: First, it has been claimed that FD can be diagnosed with MRI [4, 6]. Our study indicates this is extremely challenging due to the high variability in the tumor signal in the MR examination. Secondly, although spinal FD is considered to be rare [2], we found it to be the third most common location of the disease, affecting one out of every five patients. Thirdly, and most importantly, FD has been considered to be a benign bone tumor [2]. In our study, however, 9% of patients with FD were diagnosed as FD with a malignant transformation and in two patients, a malignant transformation was found after a previous diagnosis of FD without malignancy.

Our study has two main limitations. First, our diagnosis was based on FD patients in whom there had been a histopathological confirmation of the disease. Accordingly, the characteristics of the lesions may be concentrated towards the more complicated cases and do not necessarily represent the most typical form of FD. Nonetheless, we believe that this is not a major limitation because the results of the current study display such extensive variability in the MR appearance of the FD and this variability will continue also in those cases that have not been biopsied.

The estimated prevalence of malignant transformation has been estimated to range from 0.4 to 6.7% [8] whereas it was 9% in our study. It is probable that the potential publication bias (the probability to write and succeed in publishing a study) has increased the prevalence of FD with malignant transformation and may explain the difference in the higher prevalence of malignant transformation in our study. This same publication bias may also concern the number of FD with ABCs in our study. Interestingly, radiotherapy has been considered as a risk factor for malignant transformation in FD. In this review, however, none of the study subjects with a malignant transformation had received prior radiotherapy.

Our second study limitation is that in most of the studies there had been a poor characterization of the tumor. Only a fraction of the studies had reported the major characteristics of the tumors. For example, the sub-division to epiphyseal, metaphyseal, or diaphyseal location had been made in only 8/31 (26%), and the sub-division to centric or eccentric in 4/31 (13%) of FD lesions in long bones. The sizes of the FD lesions had been described in only 10% of all patients. Furthermore, there were no measures of either the quantitative or semiquantitative signal intensity. The reference point of SI was mentioned in only 41% of the patients. Accordingly, we are not aware of the exact meaning of hypo-, iso-, and hyperintensity SI that had been applied in the studies.

Our conclusion is that the signal intensity of FD is highly variable and thus indicates that the diagnosis of FD cannot be based on MRI alone. FD lesions may show evidence of cortical destruction and/or soft tissue extension and still be benign. FD in spine is relatively common and FD with a malignant transformation should be considered also in those patients who have not received prior radiotherapy. More studies are needed to improve the diagnostics of this relatively rare but commonly discussed bone disorder.
